# Complete mitochondrial genome of *Hemilepidotus gilberti* (Scorpaeniformes: Cottidae)

**DOI:** 10.1080/23802359.2016.1266706

**Published:** 2016-12-23

**Authors:** Young Sun Song, Il-Hun Kim, Yung Kun Kim, Ha Na Kim, Chung-Bae Kang, Won Bi Kim, Seong-Yong Kim

**Affiliations:** a National Marine Biodiversity Institute of Korea, Seocheon-gun, Chungcheongnam-do, South Korea;; b National Institute of Ecology, Seocheon-gun, Chungcheongnam-do, South Korea

**Keywords:** *Hemilepidotus gilberti*, gilbert’s Irish lord, mitochondrial genome

## Abstract

The complete mitochondrial genome of gilbert’s irish lord (*Hemilepidotus gilberti*), a fish belonging to family Cottidae, was sequenced for the first time. This complete mitochondrial genome was 16,907 nucleotides in length, consisting of 38 mitochondrial genes (13 protein-coding genes, 22 tRNA genes, 2 rRNA genes, and a control region). The order of these genes was similar to that of other teleosts. The overall A, C, G, and T nucleotide contents in mitogenome were 26.8%, 30.4%, 17.0%, and 25.8%, respectively. The A + T content (52.6%) was higher than the G + C content (47.4%). NJ phylogenetic analysis was performed for 10 related species within the family of Cottidae along with, two fish species belonging to another family (Sebastidae).

Genus *Hemilepidotus* contains six species in the world (Froese & Pauly 2016). Among them, *Hemilepidotus gilberti* belongs to the order of Scorpaeniformes and, family Cottidae (Nakabo 2013). Although many marine sculpins are known to copulate, interestingly, this species is a non-copulating sculpin (Hayakawa & Munehara 1996). They are distributed from North Pacific including eastern sea of Korea, and Hokkaido, Japan to western Bering Sea (Kim et al. 2005; Masuda et al. 1984).

In this study, a *H. gilberti* specimen was collected from a commercial fish market near the East Sea of Korea for complete mitogenome sequencing. The specimen was deposited at the National Marine Biodiversity Institute of Korea (Voucher No. MABIK PI00039348). We dissected the right dorso-lateral muscle of the specimen and preserved it in 95% ethanol. Genomic DNA was extracted from the muscle using Qiagen DNeasy Blood and Tissue kit (Qiagen Korea Ltd, Seoul, South Korea) following the manufacturer’s instructions. The complete mitochondrial DNA was sequenced on the Hiseq2000 platform using next generation sequencing technique (Illumina, San Diego, CA). Geneious 9.1.2 (Biomatters Ltd, Auckland, New Zealand), tRNA Scan-SE1.21 software (http://lowelab.ucsc.edu/tRNAScan-SE/), and MitoFish (Mitochondrial Genome Database of Fish, http://mitofish.aori.u-tokyo.ac.jp/) were used to assemble and annotate the mitochondrial DNA sequences.

The mitochondrial genome (GenBank Accession No. KX156764) was 16,907 base pairs, in length. A, C, G and T contents were 26.8%, 30.4%, 17.0% and 25.8% respectively. A–T content (52.6%) was slightly higher than G–C content (47.4%). This genome contained 13 protein-coding genes, 2 rRNA genes (12s and 16s RNA), 22 tRNA genes and 1 D-loop region. *H. gilberti* mitogenome had two rRNA subunits (12S and 16S). They were located between tRNA-Phe and tRNA-Leu (UUR) and separated by tRNA-Val gene. Among tRNAs, two forms of tRNA-Leu (UUR and CUN) and tRNA-Ser (UCN-AGN) were identified. Most genes of *H. gilberti* mitogenome were encoded on the L-strand except 16S rRNA, ND6 and tRNA (Gln, Ala, Asn, Cys, Tyr, Ser (UCN), Glu and Pro) genes which were encoded on the H-strand. All tRNAs except tRNA-Ser (AGN) have the typical clover leaf structure. The tRNA-Ser (AGN) had a reduced DHU arm. In order to discuss the phylogenetic relationships, the complete mitochondrial genome sequences of 13 species available in the public databases were used to construct a neighbor-joining (NJ) tree using the Kimura two-parameter model (Kimura 1980). Its confidence was assessed via 5000 bootstrap replications using Mega 6 (Tamura et al. 2013). The construction of phylogenetic tree showed that *H. gilbert* was clustered in the family of Cottidae and has closer relationship to the *Enophrys diceraus* and *Icelus spatula*. They were well distinguished from Family Sebastidae (Figure 1).

**Figure 1.**
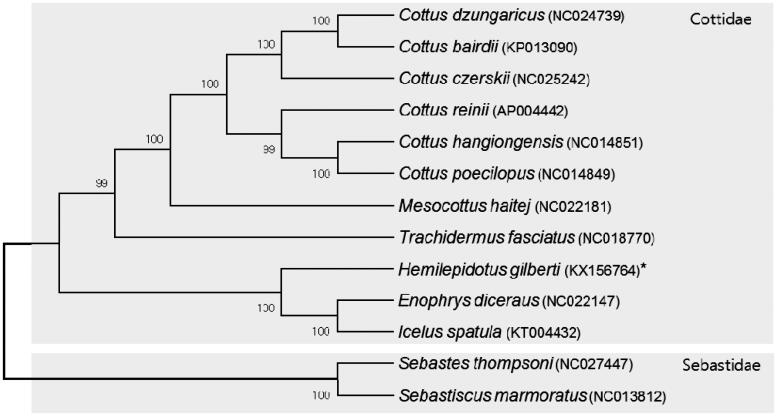
A NJ tree using the coding genes of complete mitochondrial genomes of *H. gilberti* with another 10 species belonging to family Cottidae and 2 species belonging to family Sebastidae. The complete mitogenome was downloaded from GenBank (accession number indicated after the scientific name of each species). The phylogenetic tree was constructed with MEGA6 using 5000 bootstrap replicates.
